# Clinical Characteristics of Bipolar Disorder with Comorbid Cannabis Use Disorder

**DOI:** 10.1192/j.eurpsy.2026.11211

**Published:** 2026-07-31

**Authors:** S. Ben Abderrahmen, S. Kotti

**Affiliations:** 1 Psychiatry, CHU Farhat Hached, Sousse; 2 Psychiatry, CHU Hedi Chaker, Sfax, Tunisia

## Abstract

**Introduction:**

Bipolar disorder is typically characterized by a chronic course with substantial clinical and functional burden. Among its frequent comorbidities, substance use disorders (SUD) are particularly prevalent Epidemiological studies report high rates of cannabis use among individuals with bipolar disorder, making Cannabis use disorder (CUD) highly common in this population.

**Objectives:**

This study aims o identify the clinical characteristics of bipolar disorder in cannabis users.

**Methods:**

We conducted a retrospective study of patients hospitalized in the B Psychiatry Department at Hédi Chaker Hospital in Sfax. Eligible participants were diagnosed with bipolar disorder according to DSM-5 criteria and had a comorbid diagnosis of cannabis use disorder.

**Results:**

The study included 54 patients, of whom 55% were male. Bipolar I disorder was the most frequent subtype (85%). The mean age of onset for bipolar disorder was 25 years, while cannabis use began at a mean age of 21 years. Cannabis use was reported by 55.5% of patients, with 37% using it daily. Significant associations were found between cannabis use and psychiatric hospitalization (p = 0.02), lower educational level (p = 0.013), and socioeconomic status (p = 0.02). Psychotic features were not significantly correlated with cannabis use (p = 0.275). Nearly half of the sample presented comorbid anxiety or depressive disorders. No significant correlation was observed between cannabis use and suicide attempts, nor between cannabis use and bipolar subtype (type I vs. type II).

**Conclusions:**

Cannabis use is highly prevalent among patients with bipolar disorder and is associated with earlier onset, increased hospitalization, and adverse psychosocial outcomes. These findings highlight the need for systematic screening and targeted interventions addressing cannabis use in this vulnerable population.

**Disclosure of Interest:**

None Declared

